# *N*-(5-Eth­oxy-1,3,4-thia­diazol-2-yl)benzamide

**DOI:** 10.1107/S1600536812002978

**Published:** 2012-01-31

**Authors:** Sung Kwon Kang, Nam Sook Cho, Min Kyeong Jeon

**Affiliations:** a Department of Chemistry, Chungnam National University, Daejeon 305-764, Republic of Korea

## Abstract

In the title compound, C_11_H_11_N_3_O_2_S, the dihedral angle between the thia­diazole and phenyl rings is 28.08 (7)°. In the crystal, mol­ecules are linked into an inversion dimer by a pair of inter­molecular N—H⋯N hydrogen bonds with an *R*_2_^2^(8) graph-set motif.

## Related literature

For the structures and reactivity of thia­diazole derivatives, see: Cho *et al.* (1996 ▶); Parkanyi *et al.* (1989 ▶).
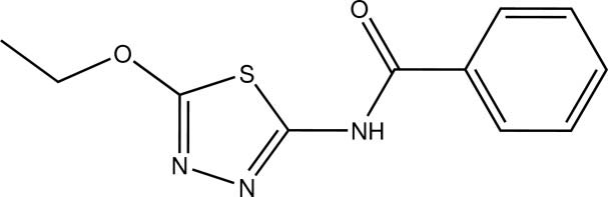


## Experimental

### 

#### Crystal data

C_11_H_11_N_3_O_2_S
*M* *_r_* = 249.29
Monoclinic, 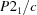

*a* = 3.9797 (5) Å
*b* = 20.138 (3) Å
*c* = 14.4305 (18) Å
β = 92.036 (2)°
*V* = 1155.8 (2) Å^3^
*Z* = 4
Mo *K*α radiation
μ = 0.27 mm^−1^
*T* = 296 K
0.27 × 0.12 × 0.11 mm

#### Data collection

Bruker SMART CCD area-detector diffractometer
Absorption correction: multi-scan (*SADABS*; Bruker, 2002 ▶) *T*_min_ = 0.956, *T*_max_ = 0.965
22225 measured reflections
2891 independent reflections
1287 reflections with *I* > 2σ(*I*)
*R*_int_ = 0.114

#### Refinement

*R*[*F* ^2^ > 2σ(*F* ^2^)] = 0.040
*wR*(*F* ^2^) = 0.081
*S* = 0.74
2891 reflections
158 parameters
H atoms treated by a mixture of independent and constrained refinement
Δρ_max_ = 0.19 e Å^−3^
Δρ_min_ = −0.19 e Å^−3^

### 

Data collection: *SMART* (Bruker, 2002 ▶); cell refinement: *SAINT* (Bruker, 2002 ▶); data reduction: *SAINT*; program(s) used to solve structure: *SHELXS97* (Sheldrick, 2008 ▶); program(s) used to refine structure: *SHELXL97* (Sheldrick, 2008 ▶); molecular graphics: *ORTEP-3* (Farrugia, 1997 ▶); software used to prepare material for publication: *WinGX* (Farrugia, 1999 ▶).

## Supplementary Material

Crystal structure: contains datablock(s) global, I. DOI: 10.1107/S1600536812002978/is5058sup1.cif

Structure factors: contains datablock(s) I. DOI: 10.1107/S1600536812002978/is5058Isup2.hkl

Supplementary material file. DOI: 10.1107/S1600536812002978/is5058Isup3.cml

Additional supplementary materials:  crystallographic information; 3D view; checkCIF report

## Figures and Tables

**Table 1:** Hydrogen-bond geometry (Å, °)

*D*—H⋯*A*	*D*—H	H⋯*A*	*D*⋯*A*	*D*—H⋯*A*
N6—H6⋯N3^i^	0.86 (2)	2.12 (2)	2.967 (3)	169 (2)

Symmetry code: (i) 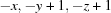.

**Fig. 1.**
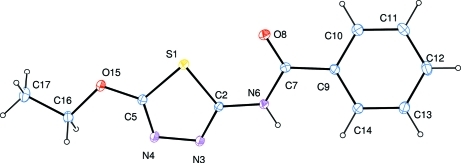
Molecular structure of the title compound, showing the atom-numbering scheme and 30% probability ellipsoids.

**Fig. 2.**
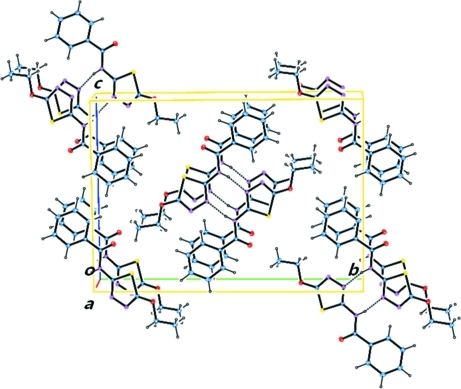
Part of the crystal structure of the title compound, showing molecules linked by intermolecular N—H···N hydrogen bonds (dashed lines).

## supplementary crystallographic information

### Comment

5-Amino-2*H*-1,2,4-thiadiazol-3-one is a five-membered ring analog of cytosine. 5-Amino-3*H*-1,3,4-thiadiazol-2-one is an isomer of 5-amino-2*H*-1,2,4-thiadiazol-3-one. The analogs of cytosine have potential to have biological activities (Parkanyi *et al.*, 1989). Thus, we attempted synthesis of derivatives of 5-amino-3*H*-1,3,4-thiadiazol-2-one (Cho *et al.*, 1996). The title compound, 2-benzoylamino-5-ethoxy-1,3,4-thiadiazole (I) is an intermediate to prepare 5-benzolamino-3*H*-1,3,4-thiadiazolin-2-one *via* hydrolysis.

The five-membered 1,3,4-thiadiazol-2-yl unit is planar, with an r.m.s. deviation of 0.003 Å from the corresponding squares plane defined by the seven constituent atoms. The bond distances of C2—N3 and C5—N4 [1.298 (2) and 1.282 (2) Å] in five-membered heterocyclic ring are shorter than those of C2—N6 and C7—N6 [1.379 (2) and 1.369 (2) Å], which is consistent with double bond character. A pair of intermolecular N6—H6···N3^i^ [symmetry code: (i) -*x*, -*y* + 1, -*z* + 1] hydrogen bonds link two molecules into an inversion dimer (Fig. 2 and Table 1), which stabilizes the crystal structure.

### Experimental

5-Amino-2-ethoxy-1,3,4-thiadiazole (1 g, 7.4 mmol) was dissolved in anhydrous dioxane (20 ml) at 80 °C. Triethylamine and benzoyl chloride (8.9 mmol) was added respectively to the above solution. The reaction solution was stirred at 80 °C for 40 minutes. TLC was used to determine the completion of the reaction. The reaction mixture was then cooled to room temperature and the ethylamine hydrochloride filtered off. The white solid was remained after the solvent was distilled off. The solid was recrystallized from ethanol to get analytical sample (product yield 55%). Colourless crystals of (I) were obtained from its ethanol solution by slow evaporation of the solvent at room temperature (m.p. 180 °C). IR (KBr, cm^-1^) 3120 (NH), 3000 (CH), 2950 (CH), 1680 (C=O), 1580 (C=N). ^-1^H NMR (DMSO-d_6_, p.p.m.): 12.5 (1*H*, b, NH), 8.3–7.4 (5*H*, m, Ph), 4.4 (2*H*, q, CH_2_), 1.4 (3*H*, t, CH_3_). ^13^C NMR (DMSO-d_6_, p.p.m.): 170.5 (amide C=O), 165.2 (O—C=N), 153.2 (C=N), 132.9, 131.6, 128.7, 128.4 (Ph), 68.2 (CH_2_), 14.4 (CH_3_). Anal. Calcd. For C_11_H_11_N_3_O_2_S: C 53.00, H 4.456, N 16.86, S 12.86. Found: C 53.07, H 4.46, N 16.83, S 13.29.

### Refinement

Atom H6 of the NH group was located in a difference Fourier map and refined freely [refined distance = 0.86 (2) Å]. Other H atoms were positioned geometrically and refined using a riding model, with C—H = 0.93–0.97 Å, and with *U*_iso_(H) = 1.2*U*_eq_(carrier C) for aromatic and methylene or 1.5*U*_eq_(carrier C) for methyl H atoms.

### Crystal data

**Table:**

C_11_H_11_N_3_O_2_S	*F*(000) = 520
*M**_r_* = 249.29	*D*_x_ = 1.433 Mg m^−^^3^
Monoclinic, *P*2_1_/*c*	Mo *K*α radiation, λ = 0.71073 Å
Hall symbol: -P 2ybc	Cell parameters from 1448 reflections
*a* = 3.9797 (5) Å	θ = 2.8–18.7°
*b* = 20.138 (3) Å	µ = 0.27 mm^−^^1^
*c* = 14.4305 (18) Å	*T* = 296 K
β = 92.036 (2)°	Needle, colourless
*V* = 1155.8 (2) Å^3^	0.27 × 0.12 × 0.11 mm
*Z* = 4	

### Data collection

**Table:**

Bruker SMART CCD area-detector diffractometer	1287 reflections with *I* > 2σ(*I*)
graphite	*R*_int_ = 0.114
φ and ω scans	θ_max_ = 28.4°, θ_min_ = 1.7°
Absorption correction: multi-scan (*SADABS*; Bruker, 2002)	*h* = −5→5
*T*_min_ = 0.956, *T*_max_ = 0.965	*k* = −26→26
22225 measured reflections	*l* = −19→19
2891 independent reflections	

### Refinement

**Table:**

Refinement on *F*^2^	Primary atom site location: structure-invariant direct methods
Least-squares matrix: full	Secondary atom site location: difference Fourier map
*R*[*F*^2^ > 2σ(*F*^2^)] = 0.040	Hydrogen site location: inferred from neighbouring sites
*wR*(*F*^2^) = 0.081	H atoms treated by a mixture of independent and constrained refinement
*S* = 0.74	*w* = 1/[σ^2^(*F*_o_^2^) + (0.0257*P*)^2^] where *P* = (*F*_o_^2^ + 2*F*_c_^2^)/3
2891 reflections	(Δ/σ)_max_ < 0.001
158 parameters	Δρ_max_ = 0.19 e Å^−^^3^
0 restraints	Δρ_min_ = −0.19 e Å^−^^3^

### Special details

**Table:**

Geometry. All e.s.d.'s (except the e.s.d. in the dihedral angle between two l.s. planes) are estimated using the full covariance matrix. The cell e.s.d.'s are taken into account individually in the estimation of e.s.d.'s in distances, angles and torsion angles; correlations between e.s.d.'s in cell parameters are only used when they are defined by crystal symmetry. An approximate (isotropic) treatment of cell e.s.d.'s is used for estimating e.s.d.'s involving l.s. planes.
Refinement. Refinement of *F*^2^ against ALL reflections. The weighted *R*-factor *wR* and goodness of fit *S* are based on *F*^2^, conventional *R*-factors *R* are based on *F*, with *F* set to zero for negative *F*^2^. The threshold expression of *F*^2^ > σ(*F*^2^) is used only for calculating *R*-factors(gt) *etc*. and is not relevant to the choice of reflections for refinement. *R*-factors based on *F*^2^ are statistically about twice as large as those based on *F*, and *R*- factors based on ALL data will be even larger.

### Fractional atomic coordinates and isotropic or equivalent isotropic displacement parameters (Å^2^)

**Table:**

	*x*	*y*	*z*	*U*_iso_*/*U*_eq_	
S1	0.39663 (15)	0.34946 (3)	0.62317 (4)	0.04682 (18)	
C2	0.2359 (5)	0.42298 (9)	0.57867 (14)	0.0375 (5)	
N3	0.2325 (5)	0.42812 (8)	0.48901 (12)	0.0467 (5)	
N4	0.3654 (5)	0.37174 (8)	0.44715 (11)	0.0473 (5)	
C5	0.4558 (5)	0.32859 (10)	0.50836 (14)	0.0411 (5)	
N6	0.1165 (5)	0.47337 (9)	0.63320 (12)	0.0433 (5)	
H6	0.021 (6)	0.5057 (12)	0.6040 (16)	0.082 (9)*	
C7	0.1183 (5)	0.46904 (10)	0.72786 (14)	0.0398 (5)	
O8	0.2192 (4)	0.41880 (7)	0.76704 (9)	0.0545 (4)	
C9	−0.0024 (5)	0.52767 (10)	0.77924 (14)	0.0399 (5)	
C10	−0.1415 (6)	0.51700 (12)	0.86450 (15)	0.0549 (7)	
H10	−0.1629	0.4739	0.8868	0.066*	
C11	−0.2486 (6)	0.56979 (13)	0.91657 (16)	0.0608 (7)	
H11	−0.3439	0.5623	0.9736	0.073*	
C12	−0.2143 (6)	0.63343 (13)	0.88413 (16)	0.0621 (7)	
H12	−0.288	0.6691	0.919	0.075*	
C13	−0.0712 (6)	0.64450 (11)	0.80031 (16)	0.0606 (7)	
H13	−0.0444	0.6877	0.7791	0.073*	
C14	0.0330 (6)	0.59171 (10)	0.74726 (15)	0.0491 (6)	
H14	0.1268	0.5994	0.6901	0.059*	
O15	0.5894 (4)	0.26919 (7)	0.49063 (10)	0.0541 (4)	
C16	0.6596 (6)	0.25697 (10)	0.39481 (14)	0.0516 (6)	
H16A	0.8359	0.2865	0.3751	0.062*	
H16B	0.4597	0.2649	0.3559	0.062*	
C17	0.7689 (6)	0.18619 (11)	0.38643 (15)	0.0639 (7)	
H17A	0.8181	0.1769	0.3231	0.096*	
H17B	0.5922	0.1574	0.4057	0.096*	
H17C	0.9666	0.1789	0.4251	0.096*	

### Atomic displacement parameters (Å^2^)

**Table:**

	*U*^11^	*U*^22^	*U*^33^	*U*^12^	*U*^13^	*U*^23^
S1	0.0621 (4)	0.0364 (3)	0.0421 (3)	0.0089 (3)	0.0035 (3)	0.0037 (3)
C2	0.0460 (14)	0.0303 (12)	0.0362 (12)	0.0008 (10)	0.0005 (10)	−0.0005 (10)
N3	0.0673 (14)	0.0351 (11)	0.0377 (11)	0.0110 (9)	0.0004 (9)	−0.0021 (9)
N4	0.0638 (14)	0.0364 (11)	0.0417 (11)	0.0106 (9)	0.0004 (9)	−0.0033 (9)
C5	0.0478 (15)	0.0334 (12)	0.0420 (13)	0.0021 (11)	0.0024 (11)	−0.0026 (11)
N6	0.0624 (14)	0.0343 (11)	0.0330 (11)	0.0088 (10)	−0.0004 (9)	0.0006 (9)
C7	0.0406 (14)	0.0376 (13)	0.0411 (13)	−0.0021 (10)	0.0011 (10)	0.0030 (11)
O8	0.0763 (12)	0.0425 (9)	0.0445 (9)	0.0106 (8)	0.0006 (8)	0.0078 (7)
C9	0.0428 (14)	0.0421 (13)	0.0348 (12)	0.0002 (11)	−0.0010 (10)	−0.0013 (11)
C10	0.0631 (18)	0.0525 (16)	0.0492 (15)	−0.0006 (13)	0.0017 (12)	−0.0016 (12)
C11	0.0647 (18)	0.0749 (19)	0.0436 (15)	−0.0008 (15)	0.0128 (12)	−0.0064 (14)
C12	0.0724 (19)	0.0667 (19)	0.0470 (16)	0.0162 (15)	−0.0007 (13)	−0.0157 (14)
C13	0.090 (2)	0.0438 (15)	0.0480 (15)	0.0117 (14)	−0.0032 (14)	−0.0036 (13)
C14	0.0667 (17)	0.0426 (14)	0.0381 (13)	0.0023 (12)	0.0013 (11)	−0.0005 (11)
O15	0.0777 (12)	0.0369 (9)	0.0483 (10)	0.0159 (8)	0.0091 (8)	−0.0015 (7)
C16	0.0564 (17)	0.0477 (15)	0.0510 (15)	0.0077 (12)	0.0056 (12)	−0.0087 (12)
C17	0.0682 (18)	0.0512 (16)	0.0720 (18)	0.0097 (13)	−0.0032 (14)	−0.0174 (13)

### Geometric parameters (Å, °)

**Table:**

S1—C2	1.727 (2)	C11—C12	1.373 (3)
S1—C5	1.733 (2)	C11—H11	0.93
C2—N3	1.298 (2)	C12—C13	1.373 (3)
C2—N6	1.379 (2)	C12—H12	0.93
N3—N4	1.399 (2)	C13—C14	1.382 (3)
N4—C5	1.282 (2)	C13—H13	0.93
C5—O15	1.337 (2)	C14—H14	0.93
N6—C7	1.368 (2)	O15—C16	1.442 (2)
N6—H6	0.86 (2)	C16—C17	1.496 (3)
C7—O8	1.220 (2)	C16—H16A	0.97
C7—C9	1.483 (3)	C16—H16B	0.97
C9—C14	1.379 (3)	C17—H17A	0.96
C9—C10	1.384 (3)	C17—H17B	0.96
C10—C11	1.378 (3)	C17—H17C	0.96
C10—H10	0.93		
			
C2—S1—C5	85.05 (10)	C10—C11—H11	120.1
N3—C2—N6	121.25 (18)	C11—C12—C13	120.1 (2)
N3—C2—S1	115.45 (15)	C11—C12—H12	120
N6—C2—S1	123.31 (16)	C13—C12—H12	120
C2—N3—N4	112.03 (16)	C12—C13—C14	120.3 (2)
C5—N4—N3	110.73 (17)	C12—C13—H13	119.8
N4—C5—O15	125.34 (19)	C14—C13—H13	119.8
N4—C5—S1	116.75 (16)	C9—C14—C13	119.9 (2)
O15—C5—S1	117.92 (15)	C9—C14—H14	120.1
C7—N6—C2	122.22 (19)	C13—C14—H14	120.1
C7—N6—H6	121.7 (16)	C5—O15—C16	115.34 (15)
C2—N6—H6	115.7 (16)	O15—C16—C17	107.87 (17)
O8—C7—N6	120.35 (19)	O15—C16—H16A	110.1
O8—C7—C9	122.38 (19)	C17—C16—H16A	110.1
N6—C7—C9	117.26 (19)	O15—C16—H16B	110.1
C14—C9—C10	119.5 (2)	C17—C16—H16B	110.1
C14—C9—C7	122.6 (2)	H16A—C16—H16B	108.4
C10—C9—C7	117.9 (2)	C16—C17—H17A	109.5
C11—C10—C9	120.4 (2)	C16—C17—H17B	109.5
C11—C10—H10	119.8	H17A—C17—H17B	109.5
C9—C10—H10	119.8	C16—C17—H17C	109.5
C12—C11—C10	119.8 (2)	H17A—C17—H17C	109.5
C12—C11—H11	120.1	H17B—C17—H17C	109.5

### Hydrogen-bond geometry (Å, °)

**Table:**

*D*—H···*A*	*D*—H	H···*A*	*D*···*A*	*D*—H···*A*
N6—H6···N3^i^	0.86 (2)	2.12 (2)	2.967 (3)	169 (2)

Symmetry codes: (i) −*x*, −*y*+1, −*z*+1.
